# A Regulatory Hierarchy Controls the Dynamic Transcriptional Response to Extreme Oxidative Stress in Archaea

**DOI:** 10.1371/journal.pgen.1004912

**Published:** 2015-01-08

**Authors:** Peter D. Tonner, Adrianne M. C. Pittman, Jordan G. Gulli, Kriti Sharma, Amy K. Schmid

**Affiliations:** 1 Computational Biology and Bioinformatics Graduate Program, Duke University, Durham, North Carolina, United States of America; 2 Biology Department, Duke University, Durham, North Carolina, United States of America; 3 Center for Systems Biology, Duke University, Durham, North Carolina, United States of America; A*STAR Microfluidics Systems Biology Lab, Singapore

## Abstract

Networks of interacting transcription factors are central to the regulation of cellular responses to abiotic stress. Although the architecture of many such networks has been mapped, their dynamic function remains unclear. Here we address this challenge in archaea, microorganisms possessing transcription factors that resemble those of both eukaryotes and bacteria. Using genome-wide DNA binding location analysis integrated with gene expression and cell physiological data, we demonstrate that a bacterial-type transcription factor (TF), called RosR, and five TFIIB proteins, homologs of eukaryotic TFs, combinatorially regulate over 100 target genes important for the response to extremely high levels of peroxide. These genes include 20 other transcription factors and oxidative damage repair genes. RosR promoter occupancy is surprisingly dynamic, with the pattern of target gene expression during the transition from rapid growth to stress correlating strongly with the pattern of dynamic binding. We conclude that a hierarchical regulatory network orchestrated by TFs of hybrid lineage enables dynamic response and survival under extreme stress in archaea. This raises questions regarding the evolutionary trajectory of gene networks in response to stress.

## Author Summary

Complex circuits of genes rather than a single gene underlie many important processes such as disease, development, and cellular damage repair. Although the wiring of many of these circuits has been mapped, how circuits operate in real time to carry out their functions is poorly understood. Here we address these questions by investigating the function of a gene circuit that responds to reactive oxygen species damage in archaea, microorganisms that represent the third domain of life. Members of this domain of life are excellent models for investigating the function and evolution of gene circuits. Components of archaeal regulatory machinery driving gene circuits resemble those of both bacteria and eukaryotes. Here we demonstrate that regulatory proteins of hybrid ancestry collaborate to control the expression of over 100 genes whose products repair cellular damage. Among these are other regulatory proteins, setting up a stepwise hierarchical circuit that controls damage repair. Regulation is dynamic, with gene targets showing immediate response to damage and restoring normal cellular functions soon thereafter. This study demonstrates how strong environmental forces such as stress may have shaped the wiring and dynamic function of gene circuits, raising important questions regarding how circuits originated over evolutionary time.

## Introduction

All organisms encounter reactive oxygen species (ROS) originating from biotic and abiotic sources. ROS are produced at relatively low levels as natural byproducts of aerobic respiration, Fenton reactions, or other biotic sources [1], [2]. In contrast, abiotic sources include environmental toxins such as solar UV radiation, pollutants, and excessive metals, which damage macromolecules [3]. In each case, oxidants must be neutralized and macromolecular damage repaired at the cellular level to enable survival. Enzymes such as superoxide dismutase and thioredoxin reductase are induced to neutralize oxidants and restore redox balance in the cell [4]. The production of these oxidant response proteins is typically transient and precisely controlled to enable rapid restoration of homeostasis following oxidant clearance and damage repair [5]. Such regulation is accomplished by a diversity of strategies throughout the microbial world. For instance, complexes of transcription factor (TF) proteins coordinate ROS-induced cell cycle block with production of repair enzymes in yeast [6]. In bacteria, TFs [7], [8] or their bound cofactors [9], [10] are directly and reversibly oxidized in the presence of ROS, altering DNA binding specificity to induce repair enzyme-coding genes [5], [11].

Relative to the other domains of life, the function of TFs that control the oxidant response in archaea remain understudied. To our knowledge, only a few transcription factors have been characterized to date [12]–[16]. Generally, components of archaeal transcription complexes are hybrid between the bacterial and eukaryal domains. For example, the basal transcriptional machinery in archaea, like that of eukaryotes, consists of transcription factor II B (Tfb), a TATA binding protein (TBP), and an RNA-Pol II-like polymerase [17]. The proteins that modulate transcription (*e.g.* stress-responsive TFs) typically resemble those of bacteria at the amino acid sequence level [18]. This class of TFs, like those of bacteria, can sense stressors or metabolites directly [14], [19], [20]. Recent evidence also suggests that these “bacterial-like” TFs can bind together on DNA combinatorially to expand their repertoire of gene regulation [21], [22]. Machine-learning efforts to reconstruct gene regulatory networks in archaea also suggest combinatorial regulation [16], [23], [24]. More generally, it remains an open question how networks of transcription factors interact dynamically to enact genome-scale regulation during stress response across the domains of life.

Here we use the salt-loving archaeon *H. salinarum* as a model, both to characterize the genome-wide binding dynamics of an ROS-responsive transcription factor, and to analyze regulatory network function during ROS stress in archaea. This hypersaline adapted archaeal model organism encounters high levels of abiotic oxidants in its natural salt lake environment, where intense solar radiation and desiccation are frequent [25]. Halophilic archaea use several complementary strategies to protect against, respond to, and repair damage induced by ROS. These include the natural protective capacity of cytoplasmic salt inclusions [26], multiple copies of repair enzymes [27], and an extensive transcription regulatory network that has been hypothesized to respond to oxidative damage [16].

However, this network was computationally inferred from gene expression data. To experimentally characterize TFs with putative involvement in this network, our previous work identified the winged helix-turn-helix DNA-binding TF RosR. This TF dynamically regulates expression of more than 300 genes in response to oxidative stress in *H. salinarum* [13]. RosR is required for survival of oxidants from multiple sources (e.g. H_2_O_2_ and paraquat). Genes directly and indirectly controlled by RosR in response to oxidant encode macromolecular repair functions. In the current study, we ask which of these genes are direct targets of RosR regulation. Integrated analysis of genome-wide binding location time course data with gene expression data demonstrates that RosR binds and regulates over 100 target genes. These encode molecular repair functions and a surprisingly high number of other TFs. RosR binds many of these sites in the absence of stress. Upon exposure to H_2_O_2_, RosR disengages from DNA at most loci. However, at other loci, RosR-DNA binding is dynamic following peroxide exposure, with locus-specific differences in TF occupancy over time. RosR binding is mediated *via* a 20-bp palindromic cis-regulatory binding sequence. Integration of data generated here in the context of other existing systems biology datasets reveals extensive combinatorial binding of RosR with multiple Tfb proteins throughout the regulon. We conclude that RosR is a master regulator of a hierarchy of TFs that performs global, dynamic physiological readjustment in response to oxidative stress.

## Results

### Conditional ChIP-chip time course experiments reveal dynamic patterns of RosR binding

**Figure 1:**
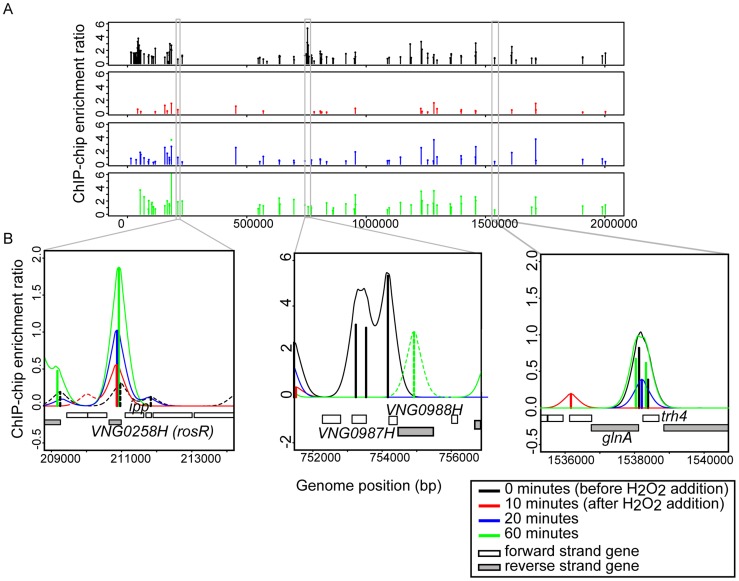
RosR binds to DNA dynamically throughout the genome in response to H_2_O_2_. (A) RosR binding peaks are plotted across the genome as a function of binding intensity. Locations of peak centers across the main chromosome are represented as vertical lines colored according to time point (see legend). Binding peak locations for the two megaplasmids are listed in S2 Table. (B) Zoom-in of selected binding regions. For each binding site shown, vertical lines represent average enrichment intensity at the binding site predicted from the bootstrapped noise estimation fits to the raw data. Surrounding curves represent model fits to the raw data. Colors for each time point are as in (A). Peaks with solid lines in each region represent those binding sites that pass statistical filtering criteria (see Methods); peaks with dotted lines do not. Gene strand designations are shown in the legend. Identification numbers or names are given for those genes immediately neighboring the binding sites.

**Figure 2:**
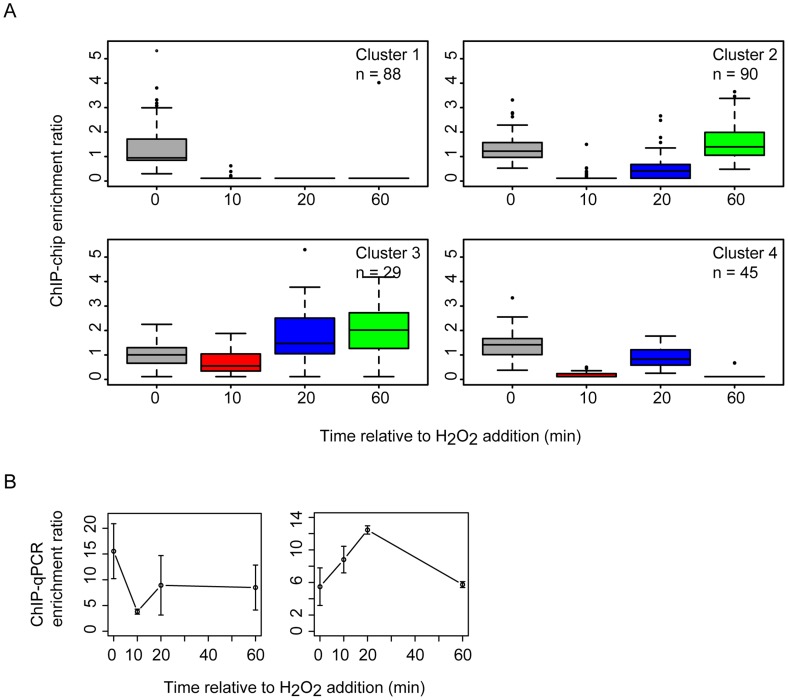
RosR occupies different promoters with four types of dynamic patterns in response to H_2_O_2_. (A) Each boxplot displays the distribution of binding enrichment across promoters in each of the four clusters. In cluster 1 (top left), RosR disengages from DNA in the presence of H_2_O_2_ and remains unbound for the duration of the time course. In cluster 2 (top right), RosR is released from DNA during H_2_O_2_ stress but re-binds within 60 minutes. In cluster 3 (bottom left), RosR binds to DNA during H_2_O_2_ stress. In cluster 4, RosR binding is dynamic and variable across the time course (bottom right). The number of genes in each cluster is indicated in the upper right corner of each boxplot. Upper and lower box borders represent the first and third quartiles, respectively. Whiskers represent the interquartile range. Black bar represents the median. Colors represent time points as in Fig. 1. (B) ChIP-qPCR validation data are shown for representative promoter regions for cluster 2 (left, *VNG0180G*) and cluster 3 (right, *VNG1732C- VNG1734H-VNG1735C* operon). Error bars represent the standard error from the mean of 9 replicate samples.

Previous work demonstrated that the RosR transcription factor is required for the differential expression of genes in response to ROS [13]. To differentiate direct from indirect targets of RosR transcriptional regulation, we mapped DNA binding locations genome-wide in the presence and absence of H_2_O_2_ over time (see Methods). A total of 189 regions (252 genes, including operons and divergently transcribed genes) were significantly enriched for RosR binding throughout the genome in the absence of stress, with fewer sites bound over time upon exposure to H_2_O_2_ (Fig. 1A, S2 Table). Upon clustering, four major RosR-DNA binding profiles were detected: (1) nearly one-third of sites (88 genes) is significantly enriched for RosR binding under standard, non-stress conditions (Fig. 1B, middle and Fig. 2A, Cluster 1). Binding enrichment at these loci fell below the statistical threshold upon the addition of H_2_O_2_ and remained low for the duration of the time course. (2) At other sites (90 genes), RosR binding was initially lost in the presence of H_2_O_2_, but binding recovered within 60 minutes (Fig. 1B, right and Fig. 2A, Cluster 2). (3) RosR binding to fewer sites (29 genes) was detectable above statistical threshold only after the addition of H_2_O_2_ and RosR remained bound to these sites for the duration of the time course (Fig. 1B, left and Fig. 2A, Cluster 3). (4) At the remainder of observed sites (45 genes), binding was more dynamic, with variability in binding enrichment throughout the time course (Fig. 2A, Cluster 4). Similar dynamic categories were observed for each of the two other genomic elements (megaplasmids) of the *H. salinarum* genome (S2 Table). Dynamic binding patterns for representative loci were validated by ChIP-qPCR as shown in Fig. 2B (cluster 2 Spearman correlation  = 0.4; cluster 3 C_s_ = 0.8). RosR binding ability in the absence of stress (clusters 1 and 4 at the 0 time point) was previously validated by ChIP-qPCR [13]. Together, these experiments suggest that RosR-DNA binding distributions are dynamic and reproducible genome-wide over time in response to oxidant treatment.

### A large fraction of RosR-DNA binding interaction dynamics is strongly associated with differential gene expression patterns

**Figure 3:**
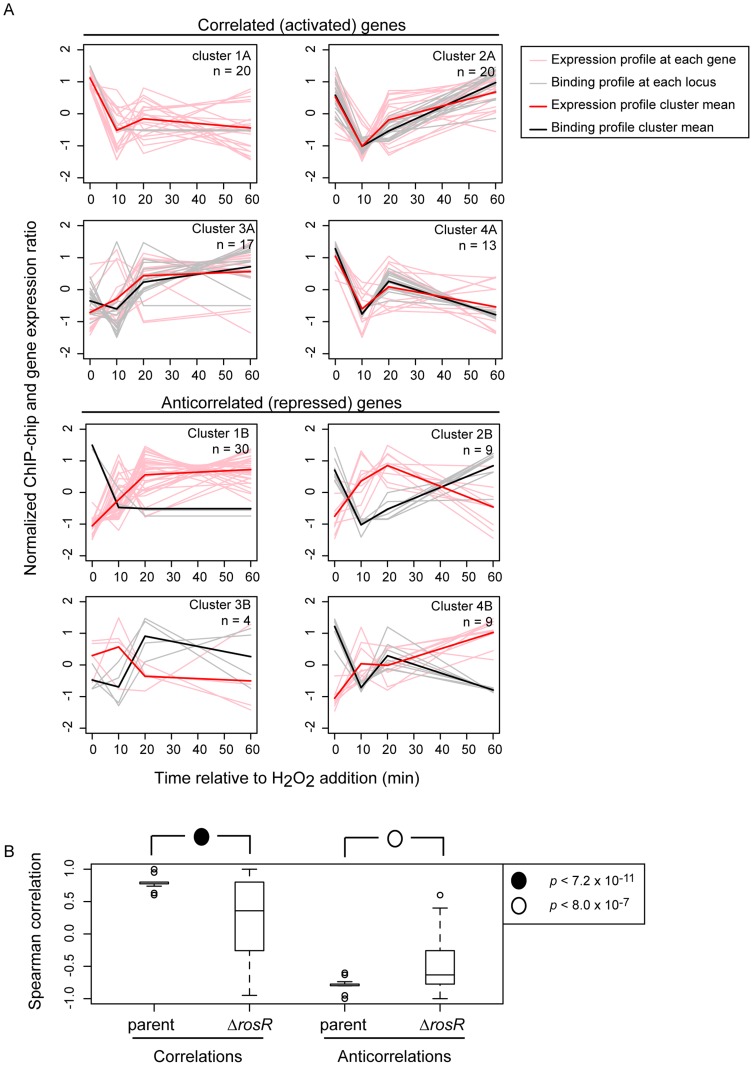
RosR is a bifunctional regulator and a large fraction of hits result in functional effects on gene expression. (A) Plots compare gene expression (pink lines) of genes nearby binding locations (grey lines) for RosR. Bolded red lines represent the mean gene expression profile in each cluster; bolded black lines represent the mean ChIP-chip binding profile. Data are mean and variance scaled. Genes with correlated gene expression and binding profiles during H_2_O_2_ stress are shown on the left (C_S_≥0.6; “A” clusters), anticorrelations right (C_S_≤−0.6; “B” clusters). The cluster designations correspond to clusters shown in Fig. 2 and number of genes in each cluster (a subset of those in Fig. 2) is indicated in the upper right corner of each graph. (B) GE-ChIP correlations trend significantly toward 0 when gene expression in the Δ*rosR* mutant background is compared to ChIP-chip profiles. Data from the same genes as in (A) make up the box plot distributions. P-values shown result from *t*-tests comparing distributions of parent to Δ*rosR* correlations (left) or anticorrelations (right).

To determine if RosR-DNA binding results in functional consequences in gene expression, we asked whether genes nearby binding loci were also differentially expressed over time. ChIP-chip binding profiles were compared to previously published gene expression data from *H. salinarum Δura3* parent vs Δ*rosR* exposed to oxidative stress over time (0, 10, 20, and 60 min relative to H_2_O_2_ addition; [13]). Of the 252 genes (including operon members) within 250 bp of a binding locus, 51 exhibit differential expression in response to H_2_O_2_ and/or deletion of *rosR* when all time points are considered together [13]. To uncover additional putative functional binding events, the correlation of RosR-DNA binding with gene expression was calculated for all 252 genes associated with binding loci. Patterns of RosR binding occupancy nearby 70 genes are strongly correlated with expression profiles (“GE-ChIP correlation”, Cs≥0.6, Fig. 3A, left graphs). Binding time course patterns at 52 other sites were anticorrelated with gene expression profiles (Cs≤−0.6, Fig. 3A, right graphs). The remaining sites were uncorrelated, which suggests that these sites represent non-specific DNA interactions and/or that other factors may be required for significant change in gene expression at these sites [28], [29]. The four clusters observed for binding profiles alone were also detected for genes exhibiting strongly correlated or anticorrelated gene expression and binding patterns (Figs. 2 and 3A). Across the distribution of strong GE-ChIP correlations and anticorrelations, deletion of *rosR* significantly alters the relationship between binding and gene expression, with a trend toward uncorrelated gene expression and binding relationships in this strain (Fig. 3B). Because the time scale of TF-DNA binding is faster than that of transcript synthesis (<1 minute vs >5 minutes, respectively; [30]), binding and expression would appear simultaneous with the resolution of the time course experiments herein (Fig. 3A). Therefore, we reasoned that the relationships between gene expression and binding profiles detected are consistent with RosR activity, with activated genes exhibiting correlated binding and expression, and repressed genes showing anticorrelated binding and expression. Together, these results suggest that: (a) dynamic binding events are strongly associated with a change in gene expression before and/or after oxidant exposure; and (b) RosR is required for direct and dynamic activation or repression of over 100 genes in response to oxidative stress in *H. salinarum*.

### Computational and experimental determination of the cis-regulatory binding sequence

**Figure 4:**
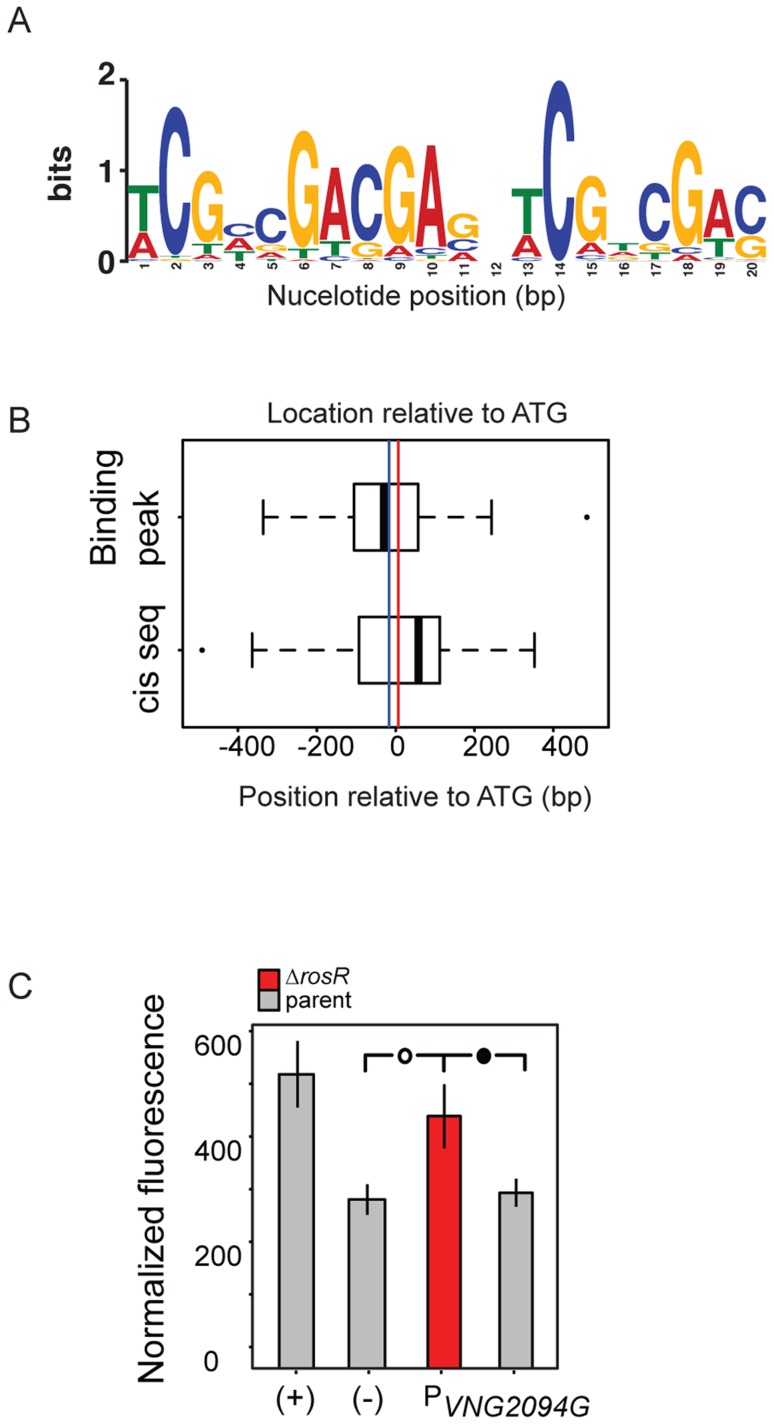
RosR binds to an imperfect palindromic cis-regulatory sequence. (A) Consensus sequence logo for the motif. Cis-regulatory sequences that make up this logo are listed in S5 Table. Information content in bits is shown on the y-axis and the residue position is given on the x-axis. (B) Position of predicted cis-regulatory sequence and ChIP-chip hits relative to start codon of genes for which a motif was detected (S5 Table). The vertical blue line indicates the average position of the binding location. The red line indicates the average position of the cis-regulatory sequence (18 bp from ATG). (C) Experimental validation of cis-regulatory motif sequence. Negative control (-) empty vector and positive control (+) with a strong constitutive promoter driving GFP expression are shown as a comparison to promoter activities of interest in each of the parent (grey bars) and Δ*rosR* (red bars) strains grown in the absence of stress. Error bars represent the standard error of the mean (minimum n = 12, at least five independent biological replicate measurements, each with 2–4 technical replicates). Filled circle within brackets represents t-test *p*<0.05, open circle *p*<0.01.

A key component of gene network function is the specific cis-regulatory binding sequence for a TF. To provide further support for RosR direct activation and repression of these target genes, we next sought to determine this binding sequence consensus for RosR. In previous work, a putative cis-regulatory sequence was computationally predicted from promoters of genes differentially expressed in response to deletion of *rosR* (direct and indirect RosR target genes; [13]). This sequence consisted of a 7 bp inverted repeat palindrome with the consensus TCGnCGA. To gain additional refinement in these predictions, the cis-regulatory sequence search was repeated using only direct RosR targets detected here by binding location analysis (S3 Table). The resultant consensus motif contained a 20 bp imperfect palindrome sequence TCGnCGACGAGnTCGnCGAC (Fig. 4A, *p*<3.5×10^−12^), which was detected nearby 37 of RosR-bound loci (∼15%; *p*<10^−37^), but not detectable elsewhere in the genome (S5 Table). Some loci contain more than one motif. Of these 37 loci with motifs detected, 40% also exhibited strong ChIP-GE associations (Cs≥|0.6|; Fig. 3). On average, motifs were located within 18 bp of ORF start sites (Fig. 4B).

To validate the function of this computationally predicted binding site experimentally, the native genomic promoter (TATA box and putative cis-regulatory sequence) of *VNG2094G* (*trh4*, a TF-coding gene) was fused to GFP. Promoter activity was assayed in the Δ*rosR* vs parent strain in the absence of stress, when RosR binding activity was evident in ChIP-chip experiments for these promoters. P*_trh4_* activity is significantly higher in the Δ*rosR* strain relative to the parent and the empty vector background control (Fig. 4C). This suggests that the predicted cis-regulatory sequence is required for RosR-mediated repression of this promoter, consistent with the genome-wide data (Figs. 1–4, S2 Table). Together, these data suggest that (a) the computationally predicted motif is biologically relevant; (b) RosR binds to the predicted cis-regulatory sequence *in vivo* to regulate gene expression; and (c) this cis-regulatory sequence carries significant importance in the function of the RosR regulatory network.

### Functional enrichment analysis of the RosR regulon

To gain additional insight into RosR function in the cell, we calculated statistical enrichment in archaeal clusters of orthologous genes functional ontology categories [31] for RosR target genes (those bound in binding location assays). These genes are significantly enriched for stress response functions (e.g. genes encoding heat shock proteins *hsp4* and *hsp5*, peroxidase *perA*), translation (e.g. genes encoding ribosomal protein), DNA replication, cell growth and division, and transcription (e.g. RNA polymerase subunits, TFIIB family member *tfbB*, LRP family homolog *trh4*; Table 1). In general, the direction of regulation corresponds with the function of these gene products. For example, genes associated with translation (e.g. *eif2B*) are downregulated upon ROS exposure, whereas stress response genes (e.g. *perA*, *hsp5*) are upregulated (S2 Table; [16]). This analysis confirms previous results implicating RosR in the regulation of genes whose products serve stress repair functions [13], but also expands the RosR regulon.

**Table 1:** Enrichment of RosR-bound targets in arCOG categories.

arCOG category	Probability	Expect	Count
Cell cycle control; cell division; chromosome partitioning	1.81E–03	2.43	7
Cell wall/membrane/envelope biogenesis	3.69E–02	3.92	7
Defense mechanisms	3.44E–02	2.52	5
Transcription	8.93E–03	13.06	21
Translation; ribosomal structure and biogenesis	1.33E–04	12.32	25
Unknown function	1.49E–03	101.32	123

### Other transcription factors required for survival under oxidative stress are major targets of RosR regulation

**Figure 5:**
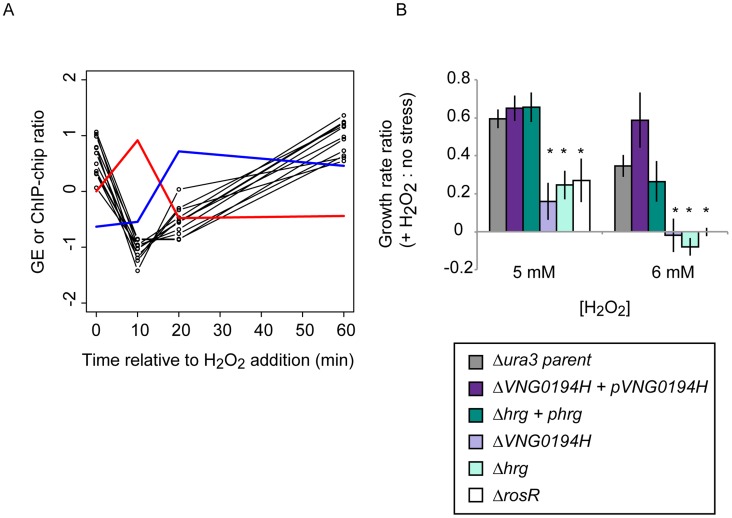
Dynamic RosR regulation of other TFs has functional consequences during ROS stress. (A) Comparison of binding and gene expression profiles of the seven of 21 TFs with cis-regulatory sequences and strong repression by RosR. Mean expression profiles are shown for TF-coding genes expressed in the early wave (red) and late wave (blue) in response to RosR binding dynamics (black lines, binding profiles for each of the 16 sites is shown). y-axis represents mean and variance scaled ChIP-chip and expression data. All expression and ChIP-chip data for these TFs are given in S2 Table. (B) RosR dynamic regulation of TFs has functional consequences. Growth rate ratios for two strains deleted of TF-coding genes (Δ*VNG0194H* and Δ*hrg*) as well as *VNG0194H* and *hrg* complemented *in trans* on a plasmid are shown. Asterisks indicate *p*-value <0.05 in t-test comparisons of growth ratios between each mutant and the parent strain. Growth rates of complemented strains are not significantly different from that of the Δ*ura3* parent strain. Raw growth data are given in S6 Table. Annotations for the strongly RosR-regulated TFs with cis-regulatory sequences are listed in Table 2, with all 21 RosR-regulated TFs listed in S3 Table.

**Figure 6:**
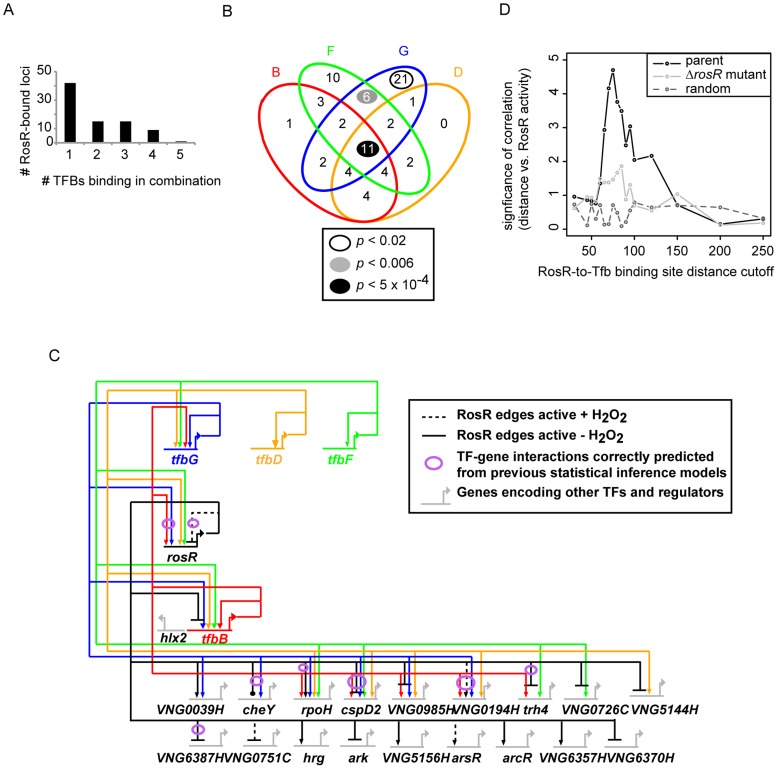
Combinatorial control of gene expression by TFIIB proteins and RosR. (A) The number of RosR-bound loci is plotted as a function of the number of different TFIIB (Tfb) proteins bound in combination. (B) Overlap between the number of sites co-bound by RosR and each Tfb protein. B, TfbB (VNG0734G, red ellipse); F, TfbF (VNG0315G, green ellipse); G, TfbG (VNG254G, blue ellipse); D, TfbD (VNG0869G, orange ellipse). *P*-values are given in the legend for significant enrichment in co-binding relative to total genome-wide RosR binding sites. Data for co-bound sites are listed in S4 Table, including TfbA data, which was omitted from the figure for clarity given the small number of co-bound RosR-TfbA sites. (C) Gene network of RosR and Tfb coordinate control of other TF-encoding genes. Tfb designations and colors are as in (B). Arrows represent activation, bars represent repression. Each edge in the network is based on both gene expression and ChIP-chip or ChIP-seq data (S2 Table, [32], [33]). (D) Comparison of RosR activity (GE-ChIP correlation) with the distance between RosR and Tfb binding loci. The negative log10 *p*-value of the significance of this comparison is given on the y-axis, and the absolute value of the distance cutoff is given on the X-axis. These correlations in the parent strain (black lines) are compared to the correlation in the Δ*rosR* mutant (grey lines) and random data with the same mean and standard deviation as the actual distribution at each distance cutoff (dark grey dotted lines).

The functional enrichment analysis revealed novel RosR targets, notably 21 genes encoding TFs and 4 other putative regulators involved in signal transduction and DNA binding (Table 1, Tables S2 and S3). Cis-regulatory sequences were detected in the vicinity of the translation start site for 14 of these TF-coding genes, including *rosR* itself (Table 2, Fig. 5, S5 Table). This could explain why direct RosR binding was not detected for many genes affected by deleting *rosR* [13] (i.e. RosR binding not detected here). For example, nearly 25% of RosR indirect gene regulation appears to be mediated through TfbB, whose encoding gene is among the TFs directly regulated by RosR (Fig. 6; [32]). Dynamic ChIP-chip profiles for seven of the 14 TF-coding genes with cis-regulatory sequences nearby were anticorrelated with their gene expression profiles (Table 2). Closer inspection of binding and gene expression profiles revealed that these seven TFs are repressed by RosR during optimum growth in the absence of stress but de-repressed in response to H_2_O_2_ (Fig. 5A). These sites were bound again within 60 minutes. Temporally coherent binding profiles resulted in two waves of time-resolved expression of TF-coding genes, with the majority of RosR-regulated TFs expressed in the late wave (Fig. 5A, S2 Table). Taken together, these results suggest that RosR regulates a hierarchy of TFs, the majority of which are transiently de-repressed in a RosR-dependent manner during oxidative stress.

**Table 2:** TFs regulated by RosR.

Gene ID*	Gene alias	arCOG function	RosR activated or repressed	cis-regulatory sequence
VNG0258H	*VNG0258H*	Predicted transcriptional regulator, PadR family	R	TTGTTGACGAGATCGTCGAG
VNG0726C	*VNG0726C*	Transcriptional regulator, TetR/AcrR family	R	TCGCCGACGAATACGGGAAG
VNG0734G	*tfbB*	Transcription initiation factor TFIIIB, Brf1 subunit/Transcription initiation factor TFIIB	R	TCGCCGACGACTACCCCGTC
VNG0735G	*hlx2*	Rec domain	R	TCGCCGACGACTACCCCGTC
VNG0751C	*VNG0751C*	Predicted transcriptional regulator, PadR family	R	ACGTCGTCGGCATCGTCGAC **
VNG0917G	*hrg*	Rec domain containing response regulator	A	TCGTCGTCGACAACGAGCAC
VNG1836G	*cspD2*	Cold shock protein, CspA family	R	ACGCAGACGACGTCGATGAC
VNG2094G	*trh4*	Transcriptional regulator, IclR family	R	TCGCCGACGACCTCGACGTG
VNG5156H	*VNG5156H*	Predicted transcriptional regulator	A	TCGAAGAGAACGACGTCGTC
VNG5182G	*arsR*	Transcriptional regulator containing HTH domain, ArsR family	A	ACTTCGACGAGTTCATCGTC
VNG6318G	*arcR*	Transcriptional regulator (IclR family)	A	ACGCGGTCGAAATCATCGAC

*TFs contain a cis-regulatory sequence, RosR activated (A) or repressed (R) according to ChIP-GE correlations.

**VNG0751C promoter contains an additional cis sequence. See S5 Table for additional sequence.

We reasoned that such TF-TF regulation might contribute to *H. salinarum* survival of extreme oxidative stress. To test this, we generated strains deleted in-frame of two of the TF-coding genes regulated by RosR (*VNG0194H* and *hrg*). Relative to the isogenic parent strain, both TF knockout strains are significantly impaired for growth in response to oxidative stress induced by addition of H_2_O_2_ to the cultures (Fig. 5B). These phenotypes are significantly complemented when the corresponding wild type copy of the TF gene is supplied *in trans* on a plasmid. These phenotypes are similar to that previously observed for the Δ*rosR* mutant strain (Fig. 5B; [13]). Together, these results implicate new TFs in oxidative stress survival in *H. salinarum*, suggest important physiological consequences for RosR regulation of other TFs, and validate hypotheses generated from systems-level datasets.

### Extensive combinatorial control of gene expression by RosR with multiple TFIIB homologs

RosR regulates many genes encoding TFs, a subset of which is required for oxidant survival. However, we reasoned that RosR might not be the only regulator of these TFs, since the phenotyping results described above are inconsistent with a classical epistatic relationship with TFs downstream of RosR in a linear regulatory cascade. To identify candidates for such co-regulation, RosR binding positions were compared to those for Tfb proteins from previously published high-resolution genome-wide DNA binding location experiments [32], [33]. Similar to RosR, Tfb binding sites are detected under standard, non-stress conditions, providing comparable physiological conditions. At 82 of each of the 252 RosR-bound loci, we also detected binding for five of seven *H. salinarum* Tfb proteins (TfbA, B, D, F, G, S4 Table). A single Tfb bound together with RosR at just over half of these loci (Fig. 6A). In contrast, 2 or more Tfbs co-bound at the same locus with RosR at 40 loci. At least four Tfbs together with RosR occupied 10 of these 40 loci (Fig. 6A, 6B). Whether the different Tfb proteins bind simultaneously or one at a time together with RosR remains unclear. While TfbA was underrepresented for co-binding with RosR, TfbG alone was significantly enriched for co-binding with RosR. At other loci, TfbF and TfbG together were enriched for co-binding with RosR (Fig. 6B). Also among the total 82 co-bound loci were 12 of the 21 RosR-regulated TF-coding genes (S4 Table, Fig. 6C).

Previous studies suggest that sequence-specific TFs in archaea activate gene expression by binding upstream of the transcription pre-initiation complex [PIC, includes TATA-binding protein (TBP) and TFIIB (Tfb)]. In contrast, most repressor TFs inhibit gene expression by binding downstream of the PIC [34]–[37]. To test this model and the mechanism of RosR gene regulation, the RosR-to-Tfb binding locus distance was calculated for the 82 RosR sites where Tfb binding was also detected (see Methods). These distances were compared to RosR activity using the GE-ChIP correlation as a proxy. Interestingly, the distance between RosR and Tfb binding loci was strongly and significantly anticorrelated with RosR activity. That is, if RosR binding upstream of Tfb is considered as a negative distance, then positive GE-ChIP correlation, or activation, is observed and *vice versa*. When these sites are binned into distance cut-offs (absolute value of 5 bp), a peak association is detected at distances of 65–75 bp (Fig. 6D). This relationship is abrogated in the Δ*rosR* mutant background (Fig. 6D, light grey trace) and is significantly different from random distributions across the distance scale (Fig. 6D, dark grey dotted trace). Together, this integrated analysis of RosR and general transcription factor networks: (a) suggests extensive and unexpected combinatorial control of gene expression between Tfb proteins and RosR; (b) provides further support for the biological significance of the GE-ChIP dynamic correlations (Fig. 3); and (c) supports the hypothesis that the relative binding position and distance between Tfb proteins and sequence-specific transcription factors dictates the activation or repression of target genes.

### Comparison of the experimentally determined RosR network to that predicted from statistical inference models

We next assessed how predictions of statistically inferred gene regulatory network models (“environmental gene regulatory influence network (EGRIN)”; [16], [23]) compared to the RosR regulatory network determined from the experiments described here. Of the 252 experimentally observed direct RosR-gene interactions, 15% were predicted from EGRIN (*p*<5.68×10^−3^; see Methods for *p*-value calculation and S2 Table for a list of genes with validated predictions). Further, the correspondence between predicted and observed target gene lists subject to combinatorial control by RosR-TfbB or RosR-TfbG was significant (*p*<2.05×10^−4^ for TfbB; *p*<2.16×10^−7^ for TfbG). In contrast, predictions from the model did not match experimental observations regarding combinatorial control by RosR-TfbD and RosR-TfbF pairs (S4 Table). Of all RosR regulated genes that were both predicted and observed, genes encoding TFs and functions in transcription are most highly enriched (arCOG category enrichment *p*<1.77×10^−5^; see also Fig. 6C). This analysis suggests that network topological predictions from the EGRIN model are accurate for RosR regulatory influences, especially for those genes that encode functions in transcriptional regulation.

## Discussion

Data and analyses presented here suggest that *H. salinarum* RosR is a bifunctional regulator that directly controls a large hierarchy of transcription factors in combination with Tfb proteins to enable extreme oxidative stress survival. The majority of these sites are bound in the absence of stress, with RosR released from DNA in the presence of oxidant. A subset of loci exhibits the opposite binding pattern. We show that RosR binds to a ∼20 bp imperfect palindrome cis-regulatory sequence and directly activates or represses genes encoding functions in transcription, macromolecular repair and central cellular physiology. We demonstrate that RosR regulates genes encoding TFs that are also required for oxidative stress survival. Such regulation is conducted in concert with Tfb proteins. We conclude that RosR plays an important role in a large transcriptional network that enables a rapid response to extreme oxidative stress followed by re-establishment of homeostasis.

The function of gene products in the RosR regulon reported here reflects the observations from our previous work [13]. Here we expand this regulon, differentiating between direct and indirect control of gene expression by RosR, including new gene targets whose products are involved in central cellular functions such as translation, transcription, and DNA replication. RosR regulation of specific genes encoding such functions is also accurately predicted from a computationally inferred gene regulatory network for *H. salinarum* [23] (S2 and S4 Tables S2; Fig. 6D). However, the RosR cis-regulatory binding sequence we detected and validated here was not predicted from the model, nor was combinatorial control of gene expression by RosR and Tfbs D and F, possibly because the inference model predicts regulatory interactions primarily based on gene expression [23]. Recent evidence suggests that such predictions can be improved by the incorporation of TF-DNA binding data (e.g. ChIP-seq or ChIP-chip, [38]). Therefore, the current work also pinpoints specific areas for model refinement.

The integrated genome-wide analysis presented here suggests hypotheses for the RosR biochemical mechanism. Dynamic TF-DNA binding analysis suggests a differential preference in RosR promoter occupancy, as some promoters are re-bound while homeostasis is restored, whereas a small subset of other sites are bound only in the presence of peroxide (Fig. 2, Cluster 3). Binding to slightly different cis-regulatory sequences could enable promoter binding under both conditions, similar to transcription factors that use Fe-S clusters as cofactors in bacteria [39]. However, we observed only one significant motif in our computational analysis (S5 Table), suggesting that other co-factors may be involved (e.g. Tfb proteins, Fig. 6). It remains unclear how and whether RosR itself senses oxidant, since no cysteines are present in the protein. Further biochemical studies are required.

In contrast to RosR targets in Cluster 3, a significant fraction of sites are bound in the absence of H_2_O_2_ and re-occupied by RosR within 60 minutes of oxidant exposure (Fig. 2, Cluster 2). Clearance of oxidant from the cell by detoxification enzymes (e.g. *perA*, *sod2*) may enable RosR to re-bind. For example, Δ*perA* mutants experience high intracellular H_2_O_2_ concentrations during mid-log phase growth, whereas H_2_O_2_ is cleared from the *H. salinarum* parent strain within the time frame tested here [16]. The gene encoding PerA is a direct target of RosR regulation (S2 and S3 Tables).

Dynamic patterns of differential promoter occupancy observed in yeast suggest that the probability of productive gene expression correlates with longer TF-DNA dwell times [40]. The addition of stress in the experiments reported here links these dynamic events to environmental perturbation. For example, TF-coding genes are found almost exclusively in dynamic binding cluster 2, which are re-bound at the earliest time point following ROS exposure (S2 Table, Fig. 2). Binding at these sites correlates well with gene expression dynamics and TF knockout strains are more sensitive to H_2_O_2_ challenge than the parent strain (Table 2, Fig. 5). The pattern of binding in cluster 2 is therefore consistent with an immediate need for TFs to work with RosR to restore homeostasis following stress exposure. Taken together, these dynamic genome-wide data point to a non-canonical mechanism for RosR regulation in response to oxidant.

Integrated analysis of several genome-wide binding location and gene expression datasets for TFIIB homologs [32], [33] with those presented here suggests a surprising degree of RosR-Tfb combinatorial control of gene expression in response to oxidant (Fig. 6). RosR combinatorial control contrasts with the *H. salinarum* nutritional regulator TrmB, which regulates far fewer TFs (only 4 for TrmB *vs*. 21 for RosR) and binds together with only one other Tfb protein at its target promoters [36]. Similarly, *H. salinarum* iron regulators Idr1 and Idr2 only regulate one other TF each [22]. Further regulatory interactions were observed between TFs, including TfbB regulation of RosR, setting up a potential feedback loop (Fig. 6D; [32]). Taken together, these data are consistent with the hypothesis that the regulatory reach of RosR under oxidative stress conditions is extended significantly *via* TF-TF network interactions.

Systems-level studies suggest that extensive TF-TF interactions may be a conserved feature of transcriptional regulation of stress response across the domains of life. For example, hierarchical regulation in response to oxidant has been observed in *Escherichia coli*, where SoxS regulates at least four other TF-coding genes (*fur*, *marA*, *marR*, *rob*; [41]), some of which in turn regulate other TF-coding genes. However, RosR control of more than 20 other TF-coding genes is closer to the order of the global nutritional regulator, CRP, which controls the expression of at least 50 other TF-coding genes. Such extensive inter-TF regulation in *H. salinarum* is also reminiscent of multi-TF regulatory networks in yeast that coordinate the cell cycle with DNA damage repair [6]. Thus, RosR appears to possess unique functional features, resembling a eukaryotic-like TF in global activation of gene expression (Fig. 1), control of a large network of TFs (Figs. 5, 6, Table 2), and extensive coordinate control of gene expression (Fig. 6; [33]). However, some features of RosR also resemble a bacterial-type TF, with its DNA binding sequence specificity (Fig. 4), repression of gene expression (Fig. 4C), and stress-specific alteration of its binding activity (Fig. 1).

## Materials and Methods

### Strains and growth conditions

Strains of *Halobacterium salinarum* NRC-1 used in this study are listed in S1 Table. Cultures were routinely grown in complex medium (CM; 250 g/L NaCl, 20 g/L MgSO_4_ 7H_2_O, 3 g/L sodium citrate, 2 g/L KCl, 10 g/L peptone). Δ*ura3*, the parent strain, and transcription factor deletion strain derivatives thereof, were grown in CM supplemented with 0.05 mg/mL uracil to complement the auxotrophy. In-frame gene deletion strains (Δ*ura3*Δ*VNG0194H, *Δ*ura3*Δ*hrg*) were constructed using the pop-in/pop-out gene deletion strategy described previously [42]. Δ*ura3*Δ*rosR*, referred to throughout as Δ*rosR* for brevity, was constructed previously [13]. *H. salinarum* strains harboring plasmids were cultured in CM supplemented with 20 µg/mL mevinolin for plasmid maintenance. H_2_O_2_ was added to mid-logarithmic phase cultures to 25 mM or at inoculation at 5 or 6 mM to test oxidative stress response as displayed in the figures.

### Dynamic, genome-wide transcription factor binding site location analysis

#### ChIP-chip data collection

*H. salinarum* harboring *VNG0258H::myc* (constructed previously in [13]) was grown to mid-logarithmic phase (OD600 ∼0.2–0.4) and either left untreated or exposed to 25 mM H_2_O_2_ for 10, 20, and 60 minutes. Transcription factor-chromatin complexes from cultures untreated and at each treated time point were then cross-linked *in vivo* with 1% formaldehyde for 30 min at room temperature and subjected to immunoprecipitation (IP) by virtue of the myc epitope tag as described previously [22]. One µg of each IP sample was hybridized against matched, mock-treated controls on a custom 2×105,000 feature 60-mer oligonucleotide microarray (Agilent Technologies). On this high-resolution array, the entire *H. salinarum* genome was tiled every 30 bp in triplicate. Randomly selected regions of the genome were spotted in quadruplicate. The custom tiling microarray design can be accessed *via* Agilent Technologies AMADID 026819, Gene Expression Omnibus (GEO) platform accession GPL18848. Dye swaps were conducted to correct for bias in incorporation. Seven biological replicate experiments were conducted for *VNG0258::myc* in the absence of H_2_O_2_ and three in the presence of H_2_O_2_. These experiments yielded a total of at least 18 replicate intensity data points per 30-bp genomic region per condition. DNA fragments were directly labeled with Cy3 and Cy5 dyes (Kreatech) as described previously [33]. Microarray slide hybridization and washing protocols were conducted according to the manufacturer's instructions (Agilent Technologies) with the exception that hybridization was conducted in the presence of 37.5% formamide at 68°C to ensure proper stringency for high G+C content of the *H. salinarum* genome (67%, [43]).

#### ChIP-chip data preprocessing, peak picking, and peak-to-gene correspondence

Resultant slides were scanned and processed with Feature Extraction software (Agilent). Raw probe intensities were first normalized within each array using density-weighted loess [40]. Second, probes were normalized to quantiles across arrays. Binding peaks were detected from normalized data for each replicate independently using MeDiChI [44]. This peak detection algorithm relies on a deconvolution-based model to determine genomic regions significantly enriched for TF binding. Binding peaks were included in subsequent correlation and statistical analyses if: (a) they were located within 250 bp of a predicted translation start site for an open reading frame (the majority of ORFs are leaderless in *H. salinarum*, see [45]); (b) the ORF was not redundant (*H. salinarum* genome encodes multiple copies of some genes; [43]); and (c) achieved *p*-value <0.05 (calculated using MeDiChI) in at least one time point. Composite *p*-values for multiple binding peaks nearby the same gene within a given time point were calculated using Fisher's combined probability test [46]. Enrichment intensity values for these combined peaks were averaged within each time point. Peaks with variable enrichment across the four time points were set to 0 intensity (i.e. no binding) at any time point that did not meet the selection criteria. This enabled comparison of ChIP-chip to gene expression data. Using this pipeline, a total of 189 binding loci were detected across the time course, which corresponded to 252 genes when experimentally determined operon members and divergently transcribed genes were included ([47]; S2 Table). Raw ChIP-chip data are available through GEO accession GSE58696.

### Detection of dynamic binding profiles in ChIP-chip data and integration with gene expression data

Time course profiles of processed ChIP-chip binding data were grouped using Spearman correlated complete linkage hierarchical clustering to identify various dynamic binding patterns. To determine the dynamic relationship between binding and gene expression, each gene in each dynamic binding cluster was correlated to expression data under the same culturing conditions as ChIP-chip from a previous study [13] (mid-logarithmic phase cultures exposed to 25 mM H_2_O_2_ at 0, 10, 20, and 60 min; GEO accession GSE33980). These correlations are referred to throughout as “GE-ChIP correlations”. GE-ChIP correlations were calculated separately for each of the Δ*rosR* deletion and isogenic parent backgrounds as an additional metric for the impact of RosR binding on gene expression. Significance of the difference in GE-ChIP correlations between the parent and Δ*rosR* strains was calculated using Student's t-test. Genes with strong GE-ChIP correlations (Cs≥0.6) were interpreted as directly activated by RosR, whereas anticorrelations (Cs≤−0.6) were interpreted as repressed. Statistical overrepresentation in archaeal clusters of orthologous genes (arCOG) functional categories [31] for RosR-bound genes was calculated for using the hypergeometric test. Enriched categories are listed in Table 1. Detailed arCOG annotations, GE-ChIP correlation values, and significance of correlations for each of the 252 genes nearby RosR binding sites are listed in S3 Table. The code repository containing the pipeline used for binding location data analysis and correspondence to gene expression can be accessed at github.com/amyschmid/rosr-chip-utils.

### Integration of data generated here with previously published systems biology datasets for *H. salinarum*

To detect RosR-Tfb combinatorial control, or “co-binding”, high resolution ChIP-chip binding data for TfbA and TfbF [33], [44] and ChIP-seq binding data for TfbB, G, and D [32] were analyzed. Genes located within 250 bp of a Tfb protein binding site with ChIP enrichment significance of *p*<0.01 were selected using the R bioconductor MeDiChI package [44]. Sites meeting the following criteria were considered to be co-bound by RosR and a Tfb protein: (a) both RosR and Tfb binding sites were detected within 250 bp of the same gene; (b) RosR and Tfb binding positions were at most 250 bp away from each other. Venn diagram was constructed using the VennDiagram package in R [48] and RosR-Tfb gene regulatory network shown in Fig. 6D was constructed using BioTapestry [49]. Distances from RosR to Tfb binding sites for each of the co-bound genes are listed in S4 Table. The relationship between Tfb-to-RosR binding site distances with RosR GE-ChIP activity values was calculated using Spearman correlation. These correlations were calculated separately for each strain background (parent and Δ*rosR*). Significance of these correlations was computed from by comparing 10,000-fold resampled data to actual data (S4 Table) at each distance cutoff in 50 bp sliding windows. The negative log10 transform of resultant *p*-values are reported. Simulated data was generated from the random normal distribution with the same mean, standard deviation, and number of samples in the actual data set (S4 Table). All other *p*-values of significance listed in the text, including comparisons to EGRIN predictions, combinatorial control, arCOG functional enrichments, etc., were calculated using the hypergeometric test against the genome-wide background distribution unless indicated otherwise.

### Validation of dynamic RosR binding profiles with ChIP-qPCR

To validate RosR binding patterns from ChIP-chip time course experiments, representative binding sites from dynamic binding pattern groups were selected. Chromatin immunoprecipitation (ChIP) samples were prepared over the time course described above and subjected to quantitative real-time PCR analysis (qPCR) using SYBR green as previously described [22], [50]. Primers used are listed in S1 Table.

### High throughput growth assays

*H. salinarum* Δ*ura3* parent, TF deletion strains Δ*ura3*Δ*VNG0194* and Δ*ura3*Δ*hrg* (deletion of *VNG0917G*), and the complementation strains (see S1 Table for strain details) were pre-grown in CM containing 0.05 mg/mL uracil (and 20 µg/mL mevinolin for complementation strains), then tested for growth phenotypes in high throughput as previously described [13]. Strains were diluted to OD600 ∼0.1 and H_2_O_2_ was added to final concentrations of 0, 5, or 6 mM. Absorbance at an optical density of 600 nm was measured every 30 minutes using the Bioscreen C (Growth Curves USA, Piscataway, NJ). Growth rates were calculated from the slope of the log2 transformed data during logarithmic growth. Reported in the figures are ratios of the growth rates of each strain under H_2_O_2_ stress relative to the same strain's growth rate without stress. All growth data are provided in S6 Table.

### Cis-regulatory sequence prediction and experimental validation

Regions of the *H. salinarum* genome sequence 250 bp upstream and downstream of each of the 189 ChIP-chip binding loci (nearby 252 genes including operons, S2 Table) were searched for a cis-regulatory consensus binding motif for RosR using MEME [51]. The output of the search was constrained to three motifs, any number of repeats per sequence, forward or reverse strand, and maximum motif width of 20 bp. Palindromic motifs were not enforced. Similar cis-regulatory sequences were detected using varying subsets of the input sequences. Motif significance was determined using the Wilcoxon signed rank test comparing randomized input sequences to actual sequences. Resultant significance of the top-scoring motif is reported in the text. Details regarding motif genomic positions, E-value of significance of similarity to consensus, and sequence are listed in S5 Table.

To validate the predicted cis-regulatory binding sequence, a 200-bp region containing the putative cis-sequence and TATA box of *VNG2094G* was cloned into the pMTF1044GFP plasmid [36], [52] by Gibson assembly [53] using primers listed in S1 Table. The maximum cloned DNA fragment size was kept to 200 bp to reduce signal from other cryptic promoter elements. *H. salinarum* Δ*ura3* parent and Δ*rosR* strains transformed with the fusion vector were grown to mid-logarithmic phase (OD600 ∼0.3–0.6) in the absence of stress in 50 mL CM. Samples were collected, washed and fixed as previously described [54] except for fixing temperature (4°C). Resultant samples were measured for fluorescence in an FLx800 fluorimeter (BioTek). Δ*ura3* harboring the empty vector (i.e. GFP-encoding gene with no promoter) or vector containing GFP-encoding gene driven by the strong constitutive *Pfdx* promoter [33] were used as negative and positive controls, respectively (S1 Table). For each strain, at least five biological replicate cultures with 2 to 4 technical replicates each were tested. Resultant raw fluorescence values were normalized to the cell density of each culture. The mean of these normalized values and standard error of the mean are presented in the figures.

## Supporting Information

S1 Table Primers and archaeal strains used in this study.
(XLS)

S2 Table Expression data and associated RosR binding sites for each gene. Contains binding location coordinates, coordinate-to-gene distances, and binding peak p-values for each gene at each time point.
(XLS)

S3 Table Detailed annotations for genes nearby RosR binding sites.
(XLS)

S4 Table Distances between RosR binding locations, each of the five TFB binding locations and associated gene identifiers.
(XLS)

S5 Table RosR cis-regulatory binding motif sequences and genome coordinates.
(XLSX)

S6 Table Raw growth data for all TF deletion strains grown under oxidative stress conditions.
(XLSX)
